# Survival in stage IV non-small cell lung cancer patients based on radiation dose to immune cells: a retrospective analysis

**DOI:** 10.3389/fonc.2025.1715751

**Published:** 2026-01-06

**Authors:** Changxing Feng, Kang Wang, Tao Hu, Fuhao Xu, Li Li, Shuanghu Yuan

**Affiliations:** 1 Department of Radiation Oncology, Shandong Cancer Hospital and Institute, Shandong First Medical University, and Shandong Academy of Medical Sciences, Jinan, Shandong, China; 2 Department of Medical Oncology, Heze Municipal Hospital, Heze, Shandong, China; 3 Department of Radiation Oncology, Anhui Provincial Cancer Hospital, Hefei, Anhui, China; 4 Department of Radiation Oncology, The First Affiliated Hospital of University of Science and Technology of China, Division of Life Sciences and Medicine, University of Science and Technology of China, Hefei, Anhui, China

**Keywords:** estimated dose of radiation to immune cells, immunology, lymphocyte nadir, stage IV NSCLC, survival

## Abstract

**Background:**

Programmed death-1 (PD-1)/programmed death-ligand 1 (PD-L1) immune checkpoint inhibitors combined with chemotherapy represent the standard first-line treatment for stage IV non-small cell lung cancer (NSCLC) without driver mutations. Both concurrent and sequential thoracic radiotherapy (RT) have been shown to improve survival outcomes. This study aimed to evaluate the prognostic significance of the estimated dose of radiation to immune cells (EDRIC) in stage IV NSCLC patients receiving first-line immunotherapy (IT), as well as the predictive performance of EDRIC in combination with inflammatory parameters for overall survival (OS).

**Methods:**

This multicenter retrospective study included 167 stage IV NSCLC patients who received concurrent or sequential RT in addition to IT. Spearman’s rank correlation was applied to assess associations between variables. Kaplan-Meier and Cox regression analyses were used to estimate OS and progression-free survival (PFS). A nomogram model was developed to evaluate the prognostic value of each parameter for OS. Receiver operating characteristic (ROC) curve analysis was performed to compare the predictive performance of the three models.

**Results:**

GTV, PTV, and N staging were positively correlated with EDRIC (r=0.3564, p<0.001; r=0.6012, p<0.001; r=0.2592, p=0.0034), whereas lymphocyte nadir was negatively correlated (r=-0.3776, p<0.001). The Cox regression analyses identified EDRIC, lymphocyte nadir, and ALI as independent prognostic factors for OS (HR = 0.42, p=0.001; HR = 2.93, p=0.001; HR = 3.04, p=0.001). EDRIC was the only independent predictor of PFS (HR = 0.43, p=0.001). The combined EDRIC-lymphocyte nadir-ALI model demonstrated superior performance compared with single-factor models in predicting 2-year and 2.5-year OS in stage IV NSCLC patients. These findings were further confirmed in an external validation cohort.

**Conclusions:**

In the IT era, higher EDRIC is associated with poorer OS and PFS, and the combination of EDRIC, lymphocyte nadir, and Advanced Lung Cancer Inflammation Index (ALI) provides more accurate prognostic assessment of OS than any single parameter alone.

## 1 Introduction

Lung cancer is the most common malignancy worldwide, with non-small cell lung cancer (NSCLC) representing the predominant histological subtype (1). Because early-stage NSCLC is often asymptomatic, most patients are diagnosed at an advanced stage with poor prognosis. With the advent of immunotherapy (IT), immune checkpoint inhibitors combined with chemotherapy have become a standard first-line treatment for advanced NSCLC (2, 3). Based on findings from the PEMBRO-RT and MDACC trials, administering concurrent or sequential thoracic radiotherapy (RT) may provide potential therapeutic benefit for patients with metastatic NSCLC (4–6).

RT can potentiate the effects of IT by inducing tumor cell apoptosis and releasing tumor-associated antigens, thereby activating antitumor immune responses. However, RT also exerts immunosuppressive effects, particularly through lymphocyte depletion (7, 8). Radiation-induced lymphocytopenia (RIL) is a recognized adverse prognostic factor in multiple solid tumors (9). Thus, quantifying the immune effects of RT is of critical importance.

Jin et al. (10) and Ladbury et al. (11) proposed and refined the estimated dose of radiation to immune cells (EDRIC), defined as the sum of equivalent uniform doses (EUD) to several organs, accounting for factors such as heart, lung, and whole-body mean dose, as well as the number of RT fractions. A retrospective study by Yu et al. (12) demonstrated that EDRIC was significantly correlated with lymphocyte depletion and served as an independent predictor of overall survival (OS) and progression-free survival (PFS) in patients with limited-stage SCLC. However, in this population, the standalone EDRIC model yielded only modest predictive performance, with a C-index of 0.617 for OS. In locally advanced NSCLC, the predictive ability of EDRIC for RIL was similarly limited, with an AUC of 0.596 (13). To improve prognostic accuracy, efforts have been made to integrate EDRIC with additional clinical indicators to develop multiparametric models.

Inflammation is a hallmark of cancer, contributing to tumor initiation, progression, and metastasis (14, 15), and is also influenced by RT and chemotherapy (16, 17). Circulating neutrophil, lymphocyte, and platelet counts reflect systemic immune-inflammatory status (18). Peripheral blood markers such as neutrophil-to-lymphocyte ratio (NLR) (19) and platelet-to-lymphocyte ratio (PLR) (20) are well-established prognostic factors in lung cancer. The Advanced Lung Cancer Inflammation Index (ALI), a composite index integrating inflammation and nutritional status, is calculated as BMI × (albumin/NLR). Several studies have demonstrated its prognostic value, with lower ALI levels associated with poor outcomes in lung cancer patients (21–24). Other biomarkers, including the systemic immune-inflammation index (SII) (25), lymphocyte-to-monocyte ratio (LMR) (26), and nutrition-based indicators such as the prognostic nutritional index (PNI) (27), have also been reported as predictors of adverse outcomes.

This study investigated the prognostic value of EDRIC and a range of inflammation- and nutrition-based indices (NLR, LMR, PLR, SII, ALI, and PNI) for OS and PFS in stage IV NSCLC patients treated with IT and RT. Furthermore, we evaluated their performance individually and in combination, aiming to develop a more accurate prognostic model for this patient population.

## 2 Materials and methods

### 2.1 Patients

This retrospective study included patients who received at least four cycles of first-line IT and thoracic RT at three centers between 2020 and 2023. Owing to the retrospective design, the requirement for informed consent was waived. A total of 167 patients with available dose measurement data were identified. Among them, 126 patients from Shandong Cancer Hospital were used as the training cohort and validation cohort, in which the prognostic model was developed and internally validated. The remaining 41 patients from Anhui Provincial Cancer Hospital and Anhui Provincial Hospital comprised an independent external validation cohort. The inclusion criteria were as follows: (1) age ≥18 years; (2) histologically confirmed NSCLC (including adenocarcinoma, squamous cell carcinoma, and large cell carcinoma); (3) clinical stage IV disease according to the AJCC TNM staging system, 8th edition; and (4) at least three complete blood counts (CBCs) obtained during thoracic RT. The exclusion criteria were: (1) prior targeted therapy for specific gene mutations (e.g., EGFR, ALK); (2) absence of detailed RT and dose measurement data; (3) active infection or ongoing corticosteroid therapy at the time of CBC assessment; and (4) concurrent or prior malignancies. This study was approved by the Ethics Committee of Shandong Cancer Hospital and Institute (ethics approval number: SDTHEC2022009020) and was conducted in accordance with the Declaration of Helsinki.

### 2.2 Data collection and EDRIC calculation

Patient data were collected by reviewing electronic medical records, including sex, age, height, weight, comorbidities (e.g., hypertension, diabetes), initial tumor pathology and stage, prior treatment history, and dates of diagnosis and disease progression. Baseline laboratory data obtained within two weeks prior to IT included serum albumin, neutrophil, platelet, lymphocyte, and monocyte counts. These values were used to calculate NLR, PLR, LMR, SII, ALI, and PNI. The EDRIC was calculated according to the methods developed by Jin et al. (10) and further refined by Ladbury et al. (11). Relevant parameters, including mean lung dose (MLD), mean heart dose (MHD), mean body dose (MBD), number of fractions, gross tumor volume (GTV), and planning target volume (PTV), were extracted from each patient’s RT treatment plan. The calculation was performed using the following formula:


$$\begin{array}{rr} EDRIC & =0.12\times MLD+0.08\times MHD\\ +\left[0.45+0.35\times 0.85\times {\left(\frac{\#of\;fractions}{45}\right)}^{\frac{1}{2}}\right]\times MBD \end{array}$$

### 2.3 Statistical analysis

The primary endpoint of this study was OS, and PFS was defined as a secondary endpoint. OS was calculated from the date of diagnosis to death from any cause or the last follow-up. PFS was defined as the interval from diagnosis to local progression or death from any cause. Optimal cutoff values for each parameter were determined using ROC curve analysis. Spearman’s rank correlation was applied to evaluate associations between EDRIC and other variables. Kaplan-Meier methods were used to estimate OS and PFS, with comparisons made using log-rank tests. Cox proportional hazards models were employed for univariate and multivariate analyses to identify factors associated with OS and PFS and to determine independent prognostic indicators. Patients were randomly assigned into a training and validation cohort at a 2:1 ratio based on independent predictors. Nomogram models predicting OS using EDRIC, ALI, and combined EDRIC-lymphocyte nadir-ALI were constructed in R software (version 4.4.2). Model performance was compared using ROC curve analysis. All statistical analyses were performed using SPSS software (version 27.0), and a p-value<0.05 was considered statistically significant.

## 3 Results

### 3.1 Baseline patient characteristics

This study included 126 patients with advanced NSCLC. The median age was 64 years (range, 36–79 years), and the cohort comprised 96 males (76.2%) and 30 females (23.8%). A history of smoking was reported in 72 patients (57.1%), and 51 patients (40.5%) had a history of alcohol consumption. Clinical staging showed that 61 patients (48.4%) were at stage IVA and 65 patients (51.6%) at stage IVB. The median follow-up was 35.5 months (range, 11.6-62.1 months). During follow-up, 57 patients (45.2%) died, and 79 patients (62.7%) experienced disease progression. The median EDRIC was 4.76 Gy (range, 0.89-12.12 Gy), and the median lymphocyte nadir during radiotherapy was 0.52 × 10^9^/L (range, 0.1-2.1 × 10^9^/L) (**Table 1**). We compared the baseline characteristics across the training, validation, and external validation cohorts, as shown in **Supplementary Table S1**. Details of the patients’ radiotherapy, including treatment intent, thoracic dose, fractionation, and doses to critical organs, are provided in **Supplementary Table S2**.

**Table 1:** Baseline characteristics of patients.

Characteristics	Total (%) (n=126)
Gender	
Male	96 (76.2)
Female	30 (23.8)
Age (y)	
(Median, range)	64 (36-79)
<65	56 (44.4)
≥65	70 (55.6)
ECOG	
0-1	118 (93.7)
≥2	8 (6.3)
Smoking history	
Yes	72 (57.1)
No	54 (42.9)
Alcohol consumption	
Yes	51 (40.5)
No	75 (59.5)
Comorbidities	
Yes	50 (39.7)
No	76 (60.3)
History	
Squamous cell carcinoma	44 (34.9)
Adenocarcinoma	78 (61.9)
Others	4 (3.2)
PDL1 Expression	
<1%	20 (15.9)
≥1%	42 (33.3)
Unknown	64 (50.8)
T stage	
T1	24 (19.0)
T2	35 (27.8)
T3	21 (16.7)
T4	46 (36.5)
N stage	
N0	12 (9.5)
N1	18 (14.3)
N2	48 (38.1)
N3	48 (38.1)
Stage	
IVA	61 (48.4)
IVB	65 (51.6)
Chemotherapy regimen	
PC	63 (50.0)
PP	18 (14.3)
TP	16 (12.7)
TC	29 (23.0)
Presence of brain metastasis	
Yes	83 (65.9)
No	43 (34.1)
Presence of liver metastasis	
Yes	68 (54.0)
No	58 (46.0)
Presence of bone metastasis	
Yes	54 (42.9)
No	72 (57.1)
PET staging	
Yes	54 (42.9)
No	72 (57.1)
Tumor location	
Lower	43 (34.1)
Middle	12 (9.6)
Upper	71 (56.3)
Location	
Left lung	57 (45.2)
Right lung	69 (54.8)
GTV (cm^3^)	
(Median, range)	48.8 (1.2-422.2)
PTV (cm^3^)	
(Median, range)	179.9 (14.8-857.5)
Lymphocyte nadir (10^9^/L)	
(Median, range)	0.52 (0.1-2.1)
EDRIC (Gy)	
(Median, range)	4.76 (0.89-12.12)

ECOG PS, Eastern Cooperative Oncology Group performance status; PC, pemetrexed plus carboplatin; PP, pemetrexed plus cisplatin; TP, paclitaxel plus cisplatin; TC, paclitaxel plus carboplatin; GTV, gross tumor volume; PTV, planning target volume; EDRIC; estimated dose of radiation to immune cells; Gy, Gray;

### 3.2 Factors affecting prognosis

ROC curves were plotted using patient survival as the state variable, and optimal cutoff values for each parameter were determined by the maximum Youden index: EDRIC = 5.63 Gy; lymphocyte nadir = 0.56 × 10^9^/L; NLR = 2.79; PLR = 143.8; LMR = 2.07; SII = 844; ALI = 306.9; PNI = 49.7. In univariate Cox regression for NSCLC, NLR, PLR, SII, LMR, ALI, lymphocyte nadir, and EDRIC were associated with OS. In multivariate analysis, ALI ≤306.9 (HR 2.64, 95% CI 1.01-6.92, P = 0.048), lymphocyte nadir ≤0.56 × 10^9^/L (HR 2.20, 95% CI 1.19-4.06, P = 0.012), and EDRIC ≤5.63 Gy (HR 0.52, 95% CI 0.29-0.91, P = 0.022) were independent prognostic factors for OS (**Table 2**). Univariate analysis also showed significant associations of NLR, PLR, SII, ALI, lymphocyte nadir, and EDRIC with PFS. Multivariate analysis identified EDRIC ≤ 5.63 Gy as an independent predictor of PFS (HR 0.50, 95% CI 0.31-0.81, P = 0.005) in NSCLC patients receiving neoadjuvant chemo-immunotherapy (**Table 3**).

**Table 2:** Cox regression analysis of overall survival.

Variable	Univariate analysis		Multivariate analysis	
	*HR (95% CI)*	*P-value*	*HR (95% CI)*	*P-value*
Age				
<60	*1.00 (Reference)*			
≥60	*1.19 (0.71-1.99)*	*0.520*		
Gender				
Male	*1.00 (Reference)*			
Female	*1.16 (0.64-2.10)*	*0.622*		
ECOG PS				
0-1	*1.00 (Reference)*			
≥2	*1.39 (0.50-3.85)*	*0.527*		
Smoking history				
Yes	*1.00 (Reference)*			
No	*0.88 (0.52-1.49)*	*0.632*		
Comorbidities				
Yes	*1.00 (Reference)*			
No	*0.77 (0.45-1.33)*	*0.349*		
History				
Squamous cell carcinoma	*1.00 (Reference)*			
Adenocarcinoma	*0.98 (0.56-1.71)*	*0.933*		
Others	*1.99 (0.59-6.72)*	*0.270*		
PDL1 Expression				
<1%	*1.00 (Reference)*			
≥1%	*1.03 (0.46-2.29)*	*0.944*		
Unknown	*1.07 (0.51-2.25)*	*0.864*		
Location				
Left lung	*1.00 (Reference)*			
Right lung	*1.68 (0.98-2.88)*	*0.059*		
T stage				
T1	*1.00 (Reference)*			
T2	*0.72 (0.33-1.58)*	0.411		
T3	*1.07 (0.45-2.52)*	0.880		
T4	*1.07 (0.52-2.21)*	0.855		
N stage				
N+	*1.00 (Reference)*			
N0	*1.31 (0.60-2.90)*	0.501		
Stage				
IVA	*1.00 (Reference)*			
IVB	*0.95 (0.57-1.60)*	*0.852*		
Chemotherapy regimen				
*PC*	*1.00 (Reference)*			
*PP*	*1.57 (0.78-3.17)*	*0.205*		
*TP*	*1.68 (0.82-3.47)*	*0.160*		
*TC*	*0.57 (0.26-1.25)*	*0.158*		
Brain metastasis				
No	*1.00 (Reference)*			
Yes	*1.13 (0.65-1.96)*	0.661		
Liver metastasis				
No	*1.00 (Reference)*			
Yes	*1.02 (0.61-1.72)*	0.943		
Bone metastasis				
No	*1.00 (Reference)*			
Yes	*1.12 (0.66-1.89)*	0.670		
Brain radiotherapy				
No	*1.00 (Reference)*			
Yes	*0.77 (0.44-1.32)*	0.336		
Liver radiotherapy				
No	*1.00 (Reference)*			
Yes	*0.64 (0.37-1.11)*	0.114		
Bone radiotherapy				
No	*1.00 (Reference)*			
Yes	*0.99 (0.57-1.74)*	0.982		
Tumor location				
Lower	*1.00 (Reference)*			
Middle	*2.18 (0.91-5.20)*	*0.079*		
Upper	*1.70 (0.91-3.16)*	*0.095*		
NLR				
> 2.79	*1.00 (Reference)*		*1.00 (Reference)*	
≤ 2.79	*0.38 (0.22-0.66)*	*0.001*	*1.17 (0.44-3.12)*	0.754
PLR				
> 143.8	*1.00 (Reference)*		*1.00 (Reference)*	
≤ 143.8	*0.53 (0.30-0.92)*	*0.023*	*1.03 (0.53-2.00)*	0.934
LMR				
> 2.07	*1.00 (Reference)*		*1.00 (Reference)*	
≤ 2.07	*2.10 (1.13-3.91)*	*0.019*	*1.21 (0.60-2.44)*	0.597
SII				
> 844	*1.00 (Reference)*		*1.00 (Reference)*	
≤ 844	*0.37 (0.22-0.63)*	0.001	*0.80 (0.36-1.75)*	0.574
PNI				
> 49.7	*1.00 (Reference)*			
≤ 49.7	*1.58 (0.93-2.71)*	0.092		
ALI				
> 306.9	*1.00 (Reference)*		*1.00 (Reference)*	
≤ 306.9	*3.04 (1.77-5.20)*	0.001	*2.64 (1.01-6.92)*	**0.048**
Lymphocyte nadir				
> 0.56×10^9^/L	*1.00 (Reference)*		*1.00 (Reference)*	
≤ 0.56×10^9^/L	*2.93 (1.64-5.23)*	*0.001*	*2.20 (1.19-4.06)*	**0.012**
EDRIC				
> 5.63Gy	*1.00 (Reference)*		*1.00 (Reference)*	
≤ 5.63Gy	*0.42 (0.25-0.71)*	*0.001*	*0.52 (0.29-0.91)*	**0.022**

HR, hazard ratio; CI, confidence interval; ECOG PS, Eastern Cooperative Oncology Group performance status; PC, pemetrexed plus carboplatin; PP, pemetrexed plus cisplatin; TP, paclitaxel plus cisplatin; TC, paclitaxel plus carboplatin; NLR, neutrophil-to-lymphocyte ratio; PLR, platelet-to-lymphocyte ratio; LMR, lymphocyte-to-monocyte ratio; SII, systemic immune-inflammation index; PNI, prognostic nutritional index; ALI, advanced lung cancer inflammation index; EDRIC, estimated dose of radiation to immune cells.

Bold values indicate statistical significance in the multivariate analysis (P < 0.05).

**Table 3:** Cox regression analysis of progression-free survival.

Variable	Univariate analysis		Multivariate analysis	
	*HR (95% CI)*	*P-value*	*HR (95% CI)*	*P-value*
Age				
<60	*1.00 (Reference)*			
≥60	*1.12 (0.72-1.74)*	*0.619*		
Gender				
Male	*1.00 (Reference)*			
Female	*1.47 (0.89-2.43)*	*0.130*		
ECOG PS				
0-1	*1.00 (Reference)*			
≥2	*1.21 (0.44-3.33)*	*0.706*		
Smoking history				
Yes	*1.00 (Reference)*			
No	*0.92 (0.59-1.45)*	*0.723*		
Comorbidities				
Yes	*1.00 (Reference)*			
No	*0.82 (0.52-1.29)*	*0.387*		
History				
Squamous cell carcinoma	*1.00 (Reference)*			
Adenocarcinoma	*0.94 (0.59-1.51)*	*0.808*		
Others	*1.18 (0.36-3.90)*	*0.784*		
PDL1 Expression				
<1%	*1.00 (Reference)*			
≥1%	*1.10 (0.56-2.18)*	*0.776*		
Unknown	*1.03 (0.54-1.96)*	*0.940*		
Location				
Left lung	*1.00 (Reference)*			
Right lung	*1.47 (0.94-2.31)*	*0.091*		
T stage				
T1	*1.00 (Reference)*			
T2	*0.78 (0.39-1.56)*	0.484		
T3	*1.18 (0.56-2.47)*	0.666		
T4	*1.30 (0.70-2.45)*	0.409		
N stage				
N+	*1.00 (Reference)*			
N0	*0.95 (0.46-1.98)*	0.897		
stage				
IVA	*1.00 (Reference)*			
IVB	*1.35 (0.86-2.10)*	*0.190*		
Chemotherapy regimen				
PC	*1.00 (Reference)*			
PP	*1.21 (0.65-2.27)*	*0.548*		
TP	*1.25 (0.66-2.39)*	*0.492*		
TC	*0.61 (0.33-1.14)*	*0.122*		
Brain metastasis				
No	*1.00 (Reference)*			
Yes	*0.99 (0.63-1.57)*	0.973		
Liver metastasis				
No	*1.00 (Reference)*			
Yes	*1.04 (0.67-1.62)*	0.854		
Bone metastasis				
No	*1.00 (Reference)*			
Yes	*0.98 (0.63-1.53)*	0.923		
Brain radiotherapy				
No	*1.00 (Reference)*			
Yes	*0.69 (0.43-1.10)*	0.119		
Liver radiotherapy				
No	*1.00 (Reference)*			
Yes	*0.77 (0.49-1.21)*	0.261		
Bone radiotherapy				
No	*1.00 (Reference)*			
Yes	*0.78 (0.48-1.28)*	0.330		
Tumor location				
Lower	*1.00 (Reference)*			
Middle	*1.33 (0.62-2.85)*	0.464		
Upper	*1.12 (0.69-1.83)*	0.656		
NLR				
> 2.79	*1.00 (Reference)*		*1.00 (Reference)*	
≤ 2.79	*0.51 (0.33-0.81)*	*0.004*	*0.96 (0.44-2.12)*	0.928
PLR				
> 143.8	*1.00 (Reference)*		*1.00 (Reference)*	
≤ 143.8	*0.42 (0.26-0.67)*	*0.001*	*0.63 (0.36-1.13)*	0.120
LMR				
> 2.07	*1.00 (Reference)*			
≤ 2.07	*1.68 (0.97-2.92)*	*0.066*		
SII				
> 844	*1.00 (Reference)*		*1.00 (Reference)*	
≤ 844	*0.41 (0.26-0.64)*	0.001	*0.72 (0.37-1.40)*	0.330
PNI				
> 49.7	*1.00 (Reference)*			
≤ 49.7	*1.26 (0.79-2.01)*	0.329		
ALI				
> 306.9	*1.00 (Reference)*		*1.00 (Reference)*	
≤ 306.9	*2.10 (1.34-3.28)*	0.001	*1.36 (0.61-3.03)*	0.450
lymphocyte nadir				
> 0.56×10^9^/L	*1.00 (Reference)*		*1.00 (Reference)*	
≤ 0.56×10^9^/L	*2.16 (1.36-3.43)*	*0.001*	*1.52 (0.96-2.50)*	0.099
EDRIC				
> 5.63Gy	*1.00 (Reference)*		*1.00 (Reference)*	
≤ 5.63Gy	*0.43 (0.27-0.67)*	*0.001*	*0.50 (0.31-0.81)*	**0.005**

HR, hazard ratio; CI, confidence interval; ECOG PS, Eastern Cooperative Oncology Group performance status; PC, pemetrexed plus carboplatin; PP, pemetrexed plus cisplatin; TP, paclitaxel plus cisplatin; TC, paclitaxel plus carboplatin; NLR, neutrophil-to-lymphocyte ratio; PLR, platelet-to-lymphocyte ratio; LMR, lymphocyte-to-monocyte ratio; SII, systemic immune-inflammation index; PNI, prognostic nutritional index; ALI, advanced lung cancer inflammation index; EDRIC, estimated dose of radiation to immune cells.

Bold values indicate statistical significance in the multivariate analysis (P < 0.05).

### 3.3 Impact of EDRIC, lymphocyte nadir, and ALI on patient prognosis

**Figure 1:**
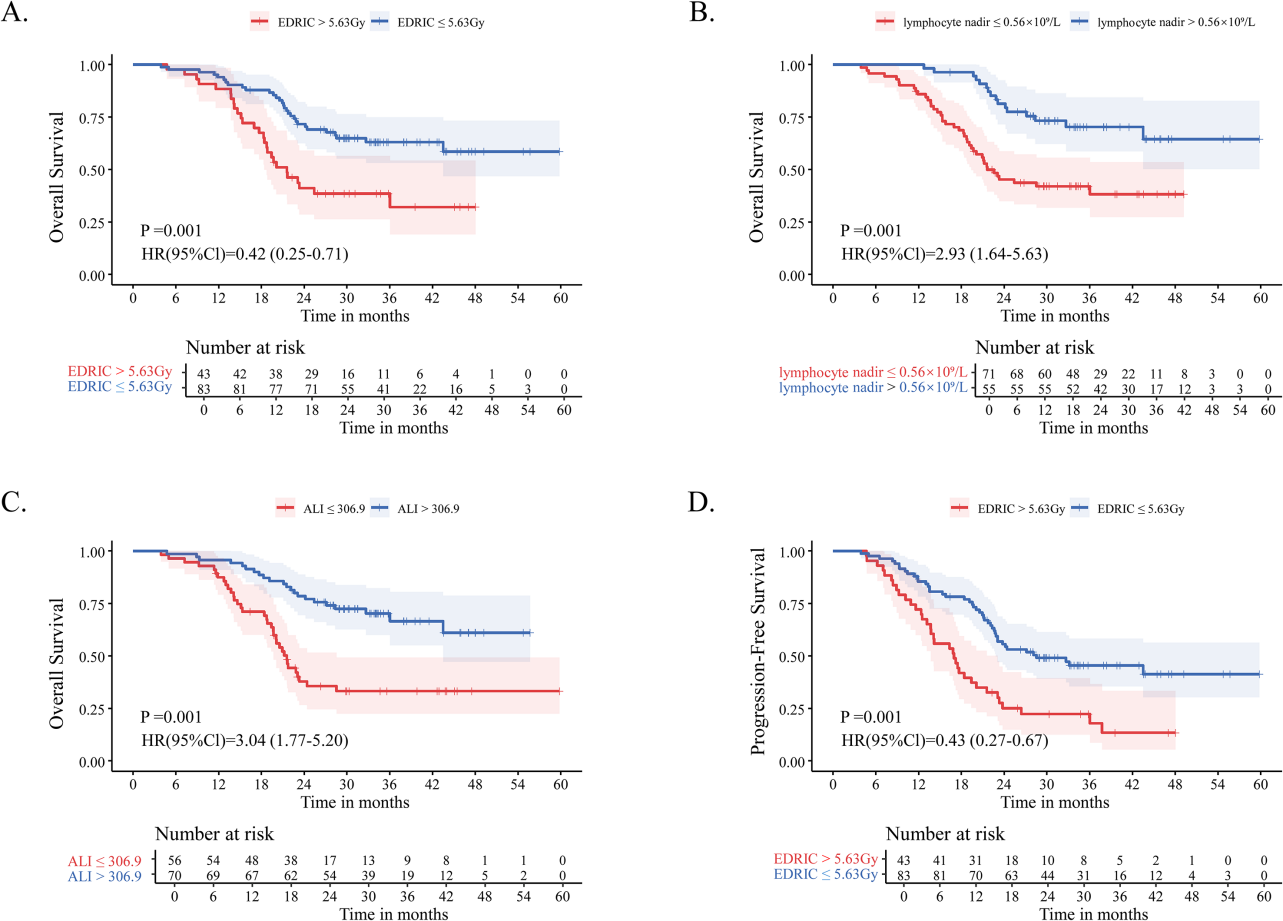
Kaplan-Meier survival curves in patients with stage IV NSCLC who received consolidative thoracic RT following first-line chemoimmunotherapy: **(A)** OS comparing the EDRIC >5.63 Gy and EDRIC ≤ 5.63 Gy groups; **(B)** OS comparing the lymphocyte nadir ≤ 0.56×10^9^/L and lymphocyte nadir >0.56×10^9^/L groups; **(C)** OS comparing the ALI ≤ 306.9 and ALI >306.9 groups; **(D)** PFS comparing the EDRIC >5.63 Gy and EDRIC ≤ 5.63 Gy groups. NSCLC, non-small cell lung cancer; RT, radiotherapy; OS, overall survival; EDRIC, estimated dose of radiation to immune cells; Gy, Gray; ALI, advanced lung cancer inflammation index; PFS, progression-free survival; HR, hazard ratio; CI, confidence interval.

Among 126 patients, 83 (65.9%) had EDRIC ≤5.63 Gy, whereas 43 (34.1%) had EDRIC >5.63 Gy. Kaplan-Meier analysis revealed significantly improved OS in patients with EDRIC ≤5.63 Gy compared with those with EDRIC >5.63 Gy. The 12-, 24-, and 36-month OS rates were 92.8%, 66.3%, and 26.5% for patients with EDRIC ≤5.63 Gy, versus 88.4%, 37.2%, and 14.0% for those with EDRIC >5.63 Gy, respectively (**Figure 1A**).

Patients with a lymphocyte nadir >0.56 × 10^9^/L exhibited significantly prolonged OS compared with those with ≤ 0.56 × 10^9^/L. The 12-, 24-, and 36-month OS rates were 100%, 76.4%, and 30.9% versus 84.5%, 40.8%, and 15.5%, respectively (**Figure 1B**). Similarly, patients with ALI >306.9 demonstrated significantly better OS compared with those with ALI ≤306.9. The 12-, 24-, and 36-month OS rates were 95.7%, 77.1%, and 27.1% versus 85.7%, 30.4%, and 16.1%, respectively (**Figure 1C**). Consistent with the OS findings, patients with EDRIC ≤5.63 Gy had significantly improved PFS compared with those with EDRIC >5.63 Gy. The 12-, 24-, and 36-month PFS rates were 84.3%, 53.0%, and 19.3% versus 70.1%, 23.3%, and 11.6%, respectively (**Figure 1D**). We also generated Kaplan–Meier curves for PFS based on lymphocyte nadir (**Supplementary Figure S1A**) and ALI (**Supplementary Figure S1B**).

**Figure 2:**
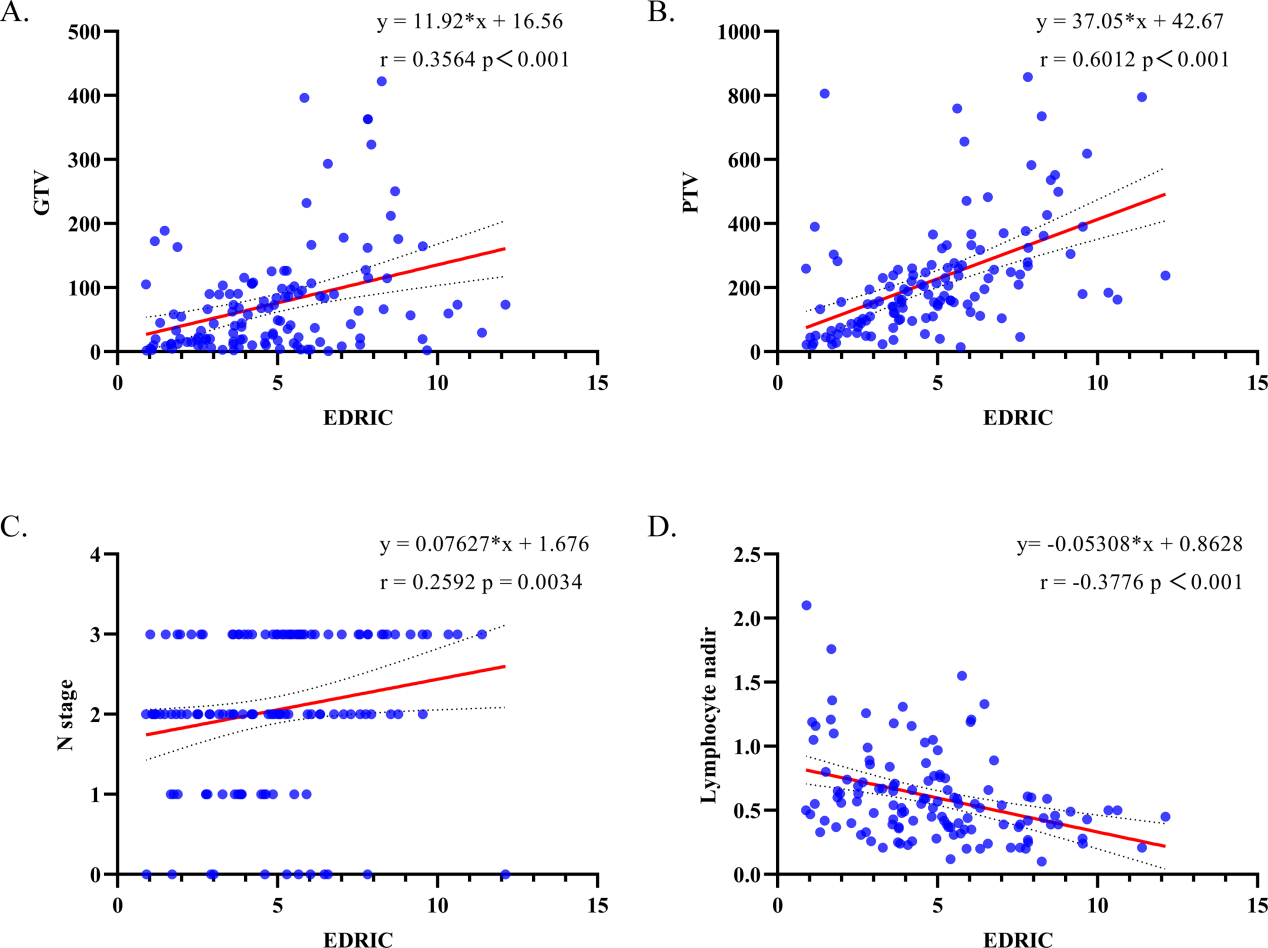
Spearman’s correlation coefficients for **(A)** GTV and EDRIC; **(B)** PTV and EDRIC; **(C)** N-stage and EDRIC; **(D)** Lymphocyte nadir and EDRIC. GTV, gross tumor volume; PTV, planning target volume; EDRIC, estimated dose of radiation to immune cells; HR, hazard ratio; CI, confidence interval; Gy, Gray;.

Spearman’s rank correlation analysis demonstrated positive correlations between EDRIC and GTV (r = 0.3564, P< 0.001; **Figure 2A**), PTV (r = 0.6012, P< 0.001; **Figure 2B**), and N stage (r = 0.2592, P = 0.0034; **Figure 2C**). In contrast, EDRIC was negatively correlated with lymphocyte nadir (r = -0.3776, P< 0.001; **Figure 2D**).

### 3.4 Comparison of nomogram prognostic models

**Figure 3:**
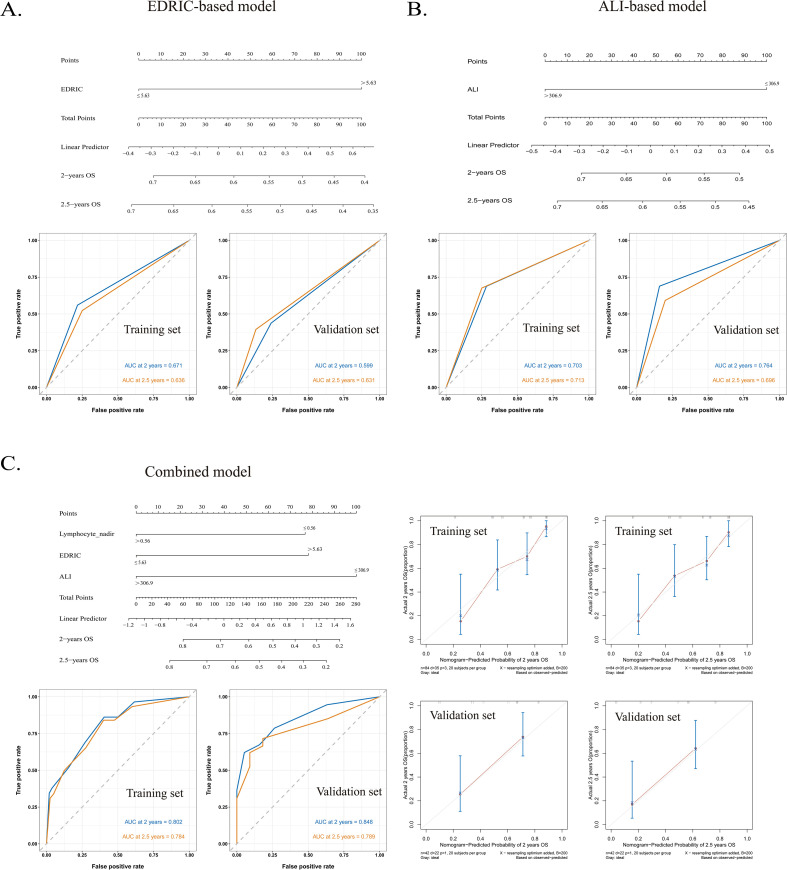
**(A)** EDRIC-based model and ROC curves for predicting OS in NSCLC patients receiving neoadjuvant chemoimmunotherapy. **(B)** ALI-based model and ROC curves for predicting OS. **(C)** Combined model and ROC curves for predicting OS, along with the calibration curve of the combined model. EDRIC, estimated dose of radiation to immune cells; ROC, receiver operating characteristic; OS, overall survival; NSCLC, non-small cell lung cancer; ALI, advanced lung cancer inflammation index.

The training cohort comprised 84 patients, while the validation cohort included 42 patients. No significant differences were observed across clinical variables between groups. The predictive performance of three nomogram models, namely EDRIC (**Figure 3A**), ALI (**Figure 3B**), and the combined EDRIC, lymphocyte nadir, and ALI model (**Figure 3C**), was evaluated, with the results summarized in **Table 4**. The calibration curve of the combined model closely aligned with the ideal reference line (**Figure 3C**).

**Table 4:** Comparison of nomogram models.

End point	Models	AUC	
		Training cohort	Validation cohort
2-year OS	Combined model	0.802	0.848
	EDRIC-based model	0.671	0.599
	ALI-based model	0.703	0.764
2.5-year OS	Combined model	0.784	0.789
	EDRIC-based model	0.636	0.631
	ALI-based model	0.713	0.696

Combined model: a combined nomogram model based on the expression of EDRIC with ALI; AUC, area under curve; OS, overall survival; EDRIC, estimated dose of radiation to immune cells; ALI, advanced lung cancer inflammation index.

Comparison of the area under the ROC curve (AUC) demonstrated that the combined model significantly outperformed the single-parameter EDRIC- and ALI-based models in predicting 2-year OS. Similar results were observed for 2.5-year OS prediction (**Table 4**).

**Figure 4:**
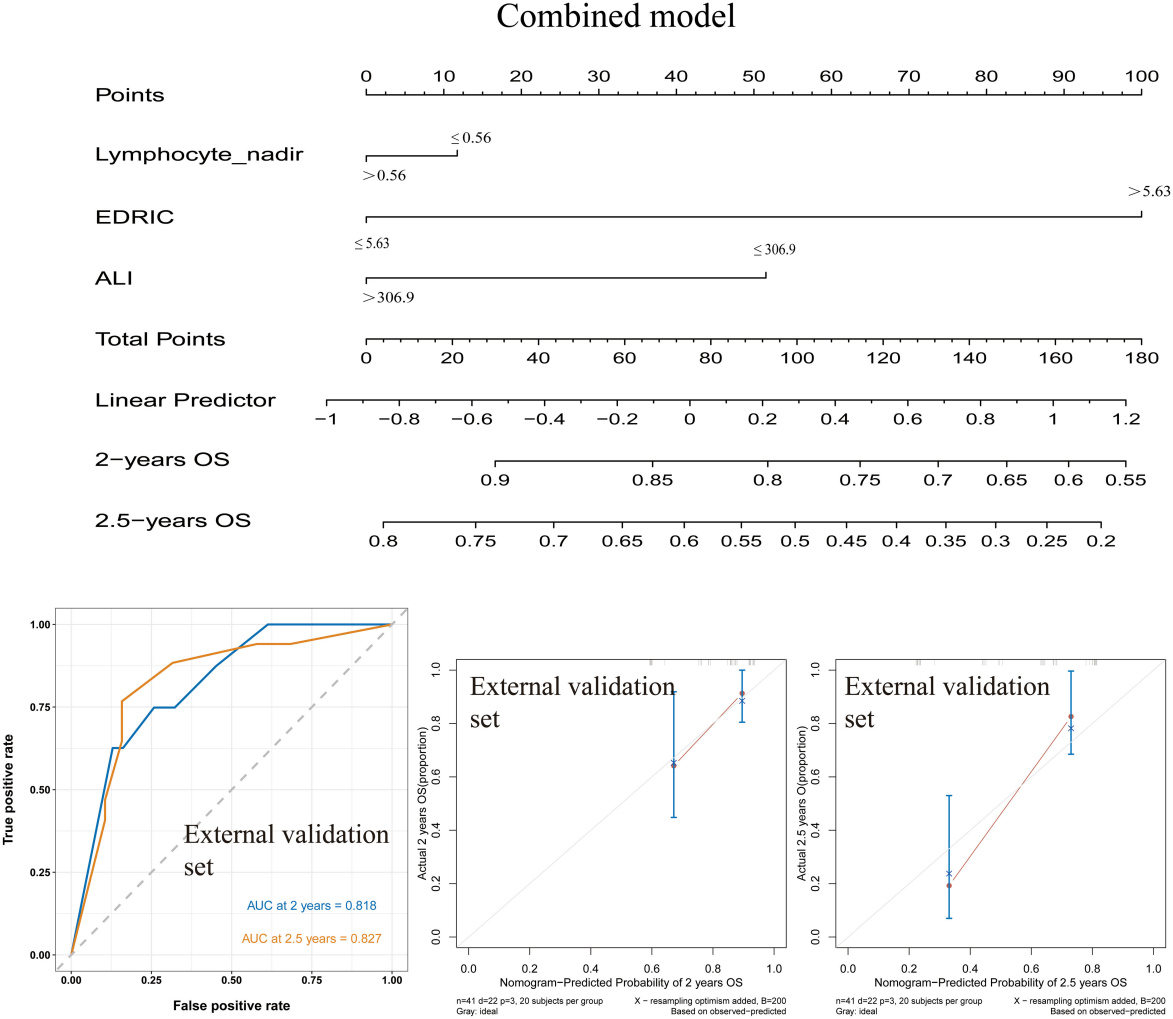
Combined model and ROC curves for predicting OS, along with the calibration curve of the combined model in the external validation cohort. ROC, receiver operating characteristic; OS, overall survival.

The combined model was further validated in an independent external cohort of 41 patients, including 26 (63.4%) with squamous cell carcinoma and 15 (36.6%) with adenocarcinoma. Within this cohort, 24 (58.5%) patients had EDRIC ≤5.63 Gy, and 19 (46.3%) had ALI >306.9. The combined model again demonstrated superior predictive ability for both 2-year and 2.5-year OS compared with the single-factor models (**Figure 4**).

Taken together, the nomogram based on EDRIC and ALI provided excellent discrimination and calibration, supporting its clinical utility in predicting survival outcomes in stage IV NSCLC patients receiving IT combined with RT.

## 4 Discussion

Based on findings from the PEMBRO-RT (4) and MDACC (5) trials, combining RT with IT has shown potential efficacy for patients with metastatic NSCLC. To our knowledge, this study is the first to investigate the prognostic value of integrating EDRIC with nutritional and inflammatory parameters in this population. Our results demonstrate that lower EDRIC scores are associated with significantly improved survival outcomes, both in terms of PFS and OS. Moreover, low baseline ALI and reduced lymphocyte nadir levels emerged as independent prognostic factors for inferior OS. While prognostic models constructed from single indicators displayed limited accuracy, the integration of EDRIC, baseline ALI, and lymphocyte nadir into a multidimensional model markedly enhanced predictive performance. This approach provides clinicians with more robust decision-making support and may facilitate the optimization of individualized treatment strategies.

In a secondary analysis of RTOG 0617, Jin et al. first proposed a model estimating the radiation dose delivered to the immune system. Following the establishment of the EDRIC model, subsequent studies confirmed that elevated EDRIC values were associated with poorer OS and PFS in stage III NSCLC (27). These findings suggest that optimizing treatment planning to minimize EDRIC could improve prognosis. Similarly, a retrospective study by Yin et al. (28) identified EDRIC as an independent prognostic factor for locally advanced NSCLC, with its impact closely correlated with GTV and N staging—results consistent with the present study. Likewise, Yang et al. (13) reported that higher EDRIC levels predicted worse OS and PFS in stage III NSCLC patients, underscoring its potential role as a biomarker for consolidation immunotherapy. These findings align closely with our results.

We also extended the analysis to the prognostic significance of ALI. A high baseline ALI was associated with longer OS, consistent with prior reports by Jafri and Mandaliya et al. (21, 29). Taken together, these findings suggest that the combined model of EDRIC, ALI, and lymphocyte nadir provides a biologically plausible and clinically useful tool for survival prediction in stage IV NSCLC.

The superior performance of the combined model may be explained by the synergistic biological effects of its components. First, lymphocytes are highly radiosensitive, with doses as low as 1 Gy substantially reducing cell survival (30, 31). Previous studies have confirmed correlations between EDRIC and peripheral lymphocyte depletion (12, 32). Since CD4+ and CD8+ T lymphocytes are critical for cytotoxic antitumor immune responses, their depletion may compromise immune surveillance and promote tumor proliferation, invasion, and metastasis (33). Second, ALI captures the interplay between systemic inflammation and nutritional status, where reduced neutrophil levels may diminish secretion of pro-angiogenic, growth, and anti-apoptotic factors, thereby limiting tumor progression (34).

Importantly, all three parameters—EDRIC, ALI, and lymphocyte nadir—are clinically simple, cost-effective, and readily available in routine practice. Once their prognostic significance is further validated, these markers could be widely implemented by oncologists worldwide, supporting clinical translation.

Nevertheless, this study has limitations. First, as a retrospective analysis, it is subject to potential selection bias and uncontrolled confounding factors. Second, EDRIC, while a promising prognostic marker, has several limitations that should be considered. It may be influenced by variations in radiotherapy planning, including target volume delineation, radiation field configuration, and dose constraints, which can differ across institutions and lead to variability in the estimated immune cell dose exposure. The EDRIC model is based on simplified assumptions and does not incorporate specific dose distributions to immune substructures such as lymph nodes or major vessels. Furthermore, although EDRIC quantifies radiation exposure to circulating immune cells, it does not capture a patient’s intrinsic immune resilience or capacity to recover from lymphocyte depletion, which may explain why combining EDRIC with host-related immune parameters (e.g., ALI or lymphocyte nadir) enhances prognostic performance. Finally, the relatively small sample size underscores the need for large-scale, multicenter, prospective trials to validate the prognostic value of EDRIC and ALI in stage IV NSCLC patients undergoing first-line immunotherapy.

## 5 Conclusions

In the era of immunotherapy, EDRIC, lymphocyte nadir, and ALI serve as independent predictors of OS in patients with stage IV NSCLC, while EDRIC alone is an independent predictor of PFS. Notably, combining EDRIC with ALI further enhances prognostic accuracy, providing a more reliable tool for outcome prediction.

## Data Availability

The raw data supporting the conclusions of this article will be made available by the authors, without undue reservation.

## Supplementary material

Supplementary Figure 1 Kaplan-Meier survival curves in patients with stage IV NSCLC who received consolidative thoracic RT following first-line chemoimmunotherapy: **(A)** PFS comparing the lymphocyte nadir ≤ 0.56×10^9^/L and lymphocyte nadir >0.56×10^9^/L groups; **(B)** PFS comparing the ALI ≤ 306.9 and ALI >306.9 groups; NSCLC, non-small cell lung cancer; RT, radiotherapy; PFS, progression-free survival; ALI, advanced lung cancer inflammation index; HR, hazard ratio; CI, confidence interval.

Supplementary Table 1 Baseline characteristics of the training, validation cohort and external validation set.

Supplementary Table 2 RT characteristics of patients, including RT intent, thoracic RT dose, fractionation, and doses to critical organs.
