# Supplementary figures and images for: Survival in stage IV non-small cell lung cancer patients based on radiation dose to immune cells: a retrospective analysis
Source: Front Oncol. 2026 Jan 6;15:1715751. doi: 10.3389/fonc.2025.1715751 (PMC12815870; doi:10.3389/fonc.2025.1715751)

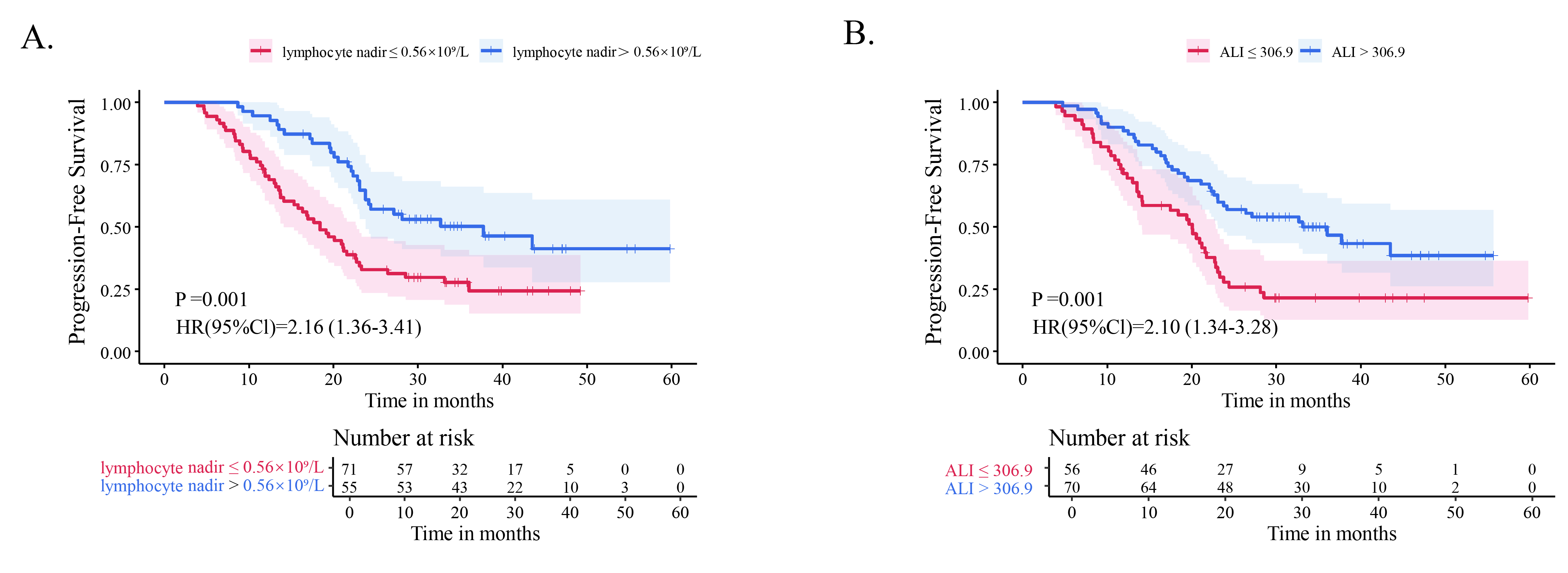

Supplement: Supplementary Figure 1 — Kaplan-Meier survival curves in patients with stage IV NSCLC who received consolidative thoracic RT following first-line chemoimmunotherapy: (A) PFS comparing the lymphocyte nadir ≤ 0.56×10^9^/L and lymphocyte nadir >0.56×10^9^/L groups; (B) PFS comparing the ALI ≤ 306.9 and ALI >306.9 groups; NSCLC, non-small cell lung cancer; RT, radiotherapy; PFS, progression-free survival; ALI, advanced lung cancer inflammation index; HR, hazard ratio; CI, confidence interval. [file Image1.tif]
