# Supplementary material for: Survival in stage IV non-small cell lung cancer patients based on radiation dose to immune cells: a retrospective analysis
Source: Front Oncol. 2026 Jan 6;15:1715751. doi: 10.3389/fonc.2025.1715751 (PMC12815870; doi:10.3389/fonc.2025.1715751)
Supplement: Supplementary Table 1 — Baseline characteristics of the training, validation cohort and external validation set. [file Table1.docx]

**Table S1. Baseline characteristics of the training, validation cohort and external validation set**

| Characteristics | training cohort | validation cohort | external validation set |
| --- | --- | --- | --- |
|  | Total (%) (n=84) | Total (%) (n=42) | Total (%) (n=41) |
| Gender  Male  Female | 60 (71.4)  24 (28.6) | 36 (85.7)  6 (14.3) | 28 (68.3)  13 (31.7) |
| Age (y)  (Median, range)  <65  ≥65 | 63 (38-79)  37 (45.1)  47 (54.9) | 61 (36-75)  19 (45.2)  23 (54.8) | 61 (54-74)  24 (58.5)  17 (41.5) |
| ECOG PS  0-1  ≥2 | 79 (94.0)  5 (6.0) | 39 (92.9)  3 (7.1) | 38 (92.7)  3 (7.3) |
| Smoking history  Yes  No | 46 (54.8)  38 (45.2) | 27 (64.3)  15 (35.7) | 26 (63.4)  15 (36.6) |
| Alcohol consumption  Yes  No  Comorbidities  Yes  No  History  Squamous cell carcinoma  Adenocarcinoma  Others | 33 (39.3)  51 (60.7)  31 (36.9)  53 (63.1)  27 (32.1)  54 (64.3)  3 (3.6) | 18 (42.9)  24 (57.1)  19 (45.2)  23 (54.8)  17 (40.5)  24 (57.1)  1 (2.4) | 23 (56.1)  18 (43.9)  19 (46.3)  22 (53.7)  12 (29.3)  28 (68.3)  1 (2.4) |
| PDL1 Expression  <1%  ≥1%  Unknown | 13 (15.5)  28 (33.3)  43 (51.2) | 7 (16.7)  14 (33.3)  21 (50.0) | 7 (17.4)  10 (24.1)  24 (58.5) |
| T stage  T1  T2  T3  T4 | 16 (19.0)  22 (26.2)  16 (19.0)  30 (35.8) | 8 (19.0)  13 (31.0)  5 (11.9)  16 (38.1) | 9 (22.3)  11 (26.8)  10 (24.1)  11 (26.8) |
| N stage  N0  N1  N2  N3 | 8 (9.5)  11 (13.1)  32 (38.1)  33 (39.3) | 4 (9.5)  7 (16.7)  16 (38.1)  15 (35.7) | 6 (14.7)  7 (17.1)  14 (34.1)  14 (34.1) |
| stage  IVA  IVB | 40 (47.6)  44 (52.4) | 21 (50.0)  21 (50.0) | 18 (43.9)  23 (56.1) |
| Chemotherapy regimen  PC  PP  TP  TC | 44 (52.4)  12 (14.3)  10 (11.9)  18 (21.4) | 19 (45.2)  6 (14.3)  6 (14.3)  11 (26.2) | 22 (53.7)  3 (7.3)  7 (17.1)  9 (21.9) |
| Presence of brain metastasis  Yes  No | 62 (73.8)  22 (26.2) | 21 (50.0)  21 (50.0) | 23 (56.1)  18 (43.9) |
| Presence of liver metastasis  Yes  No | 45 (53.6)  39 (46.4) | 23 (54.8)  19 (45.2) | 22 (53.7)  19 (46.3) |
| Presence of bone metastasis  Yes  No | 31 (36.9)  53 (63.1) | 23 (54.8)  19 (45.2) | 14 (34.1)  27 (65.9) |
| PET staging  Yes  No  Tumor location  Lower  Middle  Upper  Location  Left lung  Right lung | 37 (44.0)  47 (56.0)  33 (39.3)  9 (10.7)  42 (50.0)  37 (44.0)  47 (56.0) | 17 (40.5)  25 (59.5)  29 (69.0)  3 (7.2)  10 (23.8)  20 (47.6)  22 (52.4) | 15 (36.6)  26 (63.4)  18 (43.9)  6 (14.6)  17 (41.5)  19 (46.3)  22 (53.7) |
| GTV (cm^3^)  (Median, range)  PTV (cm^3^)  (Median, range) | 42.4 (2.1-396.3)  309.02 (14.8-857.5) | 68.1 (1.2-422.2)  219.9 (19.2-805.9) | 48.8 (2.4-386.4)  179.9 (36.5-653.1) |
| lymphocyte nadir (10⁹/L)  (Median, range)  EDRIC (Gy)  (Median, range) | 0.56 (0.12-1.76)  4.73 (0.89-12.12) | 0.53 (0.42-0.71)  4.78 (0.9-11.39) | 0.56 (0.1-1.7)  4.82 (0.89-11.77) |

ECOG PS, Eastern Cooperative Oncology Group performance status; PC, pemetrexed plus carboplatin; PP, pemetrexed plus cisplatin; TP, paclitaxel plus cisplatin; TC, paclitaxel plus carboplatin; GTV, gross tumor volume; PTV, planning target volume; EDRIC; estimated dose of radiation to immune cells; Gy, Gray;
