# Supplementary material for: Survival in stage IV non-small cell lung cancer patients based on radiation dose to immune cells: a retrospective analysis
Source: Front Oncol. 2026 Jan 6;15:1715751. doi: 10.3389/fonc.2025.1715751 (PMC12815870; doi:10.3389/fonc.2025.1715751)
Supplement: Supplementary Table 2 — RT characteristics of patients, including RT intent, thoracic RT dose, fractionation, and doses to critical organs. [file Table2.docx]

**Table S2. RT characteristics of patients, including RT intent, thoracic RT dose, fractionation, and doses to critical organs**

| ***RT intent*** | ***thoracic RT dose (Gy)*** | ***fractionation*** | **MHD *(cGy)*** | **MLD *(cGy)*** |
| --- | --- | --- | --- | --- |
| *consolidation* | 55 | 22 | 594.4 | 1327.3 |
| *palliative thoracic RT* | 44 | 22 | 1870 | 800.4 |
| *consolidation* | 60 | 24 | 292.9 | 1057.8 |
| *consolidation* | 54 | 18 | 40.4 | 538.3 |
| *consolidation* | 50 | 20 | 1075.3 | 931.3 |
| *consolidation* | 51 | 17 | 112.3 | 991.2 |
| *consolidation* | 56 | 7 | 11.7 | 287.9 |
| *consolidation* | 60 | 30 | 87.8 | 747.4 |
| *consolidation* | 56 | 8 | 742.1 | 295.6 |
| *consolidation* | 60.2 | 28 | 1551.3 | 1243.5 |
| *consolidation* | 60 | 30 | 1238.5 | 1121.8 |
| *consolidation* | 59 | 32 | 2504 | 1627.2 |
| *consolidation* | 54 | 27 | 361.1 | 1041.8 |
| *consolidation* | 50 | 10 | 425.8 | 384.1 |
| *consolidation* | 60 | 30 | 164.3 | 812 |
| *consolidation* | 45 | 15 | 494.1 | 1027.7 |
| *consolidation* | 45 | 15 | 26.2 | 335.4 |
| *consolidation* | 60 | 20 | 173 | 429.9 |
| *consolidation* | 60 | 30 | 97.4 | 952.6 |
| *consolidation* | 66 | 30 | 2104.9 | 1126.6 |
| *palliative thoracic RT* | 45 | 15 | 253.4 | 688.2 |
| *consolidation* | 45 | 15 | 82.8 | 692.6 |
| *consolidation* | 50 | 25 | 71.8 | 1121.5 |
| *consolidation* | 60 | 20 | 538 | 462.3 |
| *consolidation* | 50 | 25 | 2123.1 | 1119.4 |
| *consolidation* | 45 | 15 | 110.2 | 254.3 |
| *consolidation* | 54 | 27 | 296.2 | 466.6 |
| *consolidation* | 60 | 30 | 1494.1 | 1345 |
| *consolidation* | 55.1 | 37 | 325.4 | 784.2 |
| *consolidation* | 55 | 22 | 270.8 | 504.7 |
| *consolidation* | 54 | 27 | 1673.4 | 833.4 |
| *consolidation* | 50 | 25 | 572.7 | 652 |
| *consolidation* | 58.2 | 34 | 640.1 | 983 |
| *consolidation* | 60 | 27 | 319.3 | 740.6 |
| *consolidation* | 50.4 | 28 | 543.6 | 953.6 |
| *consolidation* | 50.4 | 28 | 594.4 | 1327.3 |
| *consolidation* | 54 | 27 | 563.9 | 947.7 |
| *palliative thoracic RT* | 36 | 18 | 554.7 | 630.7 |
| *consolidation* | 54 | 27 | 1101.5 | 1422.2 |
| *palliative thoracic RT* | 45 | 15 | 800.9 | 741.7 |
| *consolidation* | 51 | 17 | 444.9 | 868 |
| *consolidation* | 51 | 17 | 943.4 | 799.3 |
| *consolidation* | 60 | 30 | 913.3 | 1004.8 |
| *consolidation* | 58 | 29 | 63.3 | 593.2 |
| *consolidation* | 58 | 24 | 145.4 | 948.9 |
| *consolidation* | 61.6 | 28 | 88.7 | 712.7 |
| *consolidation* | 60 | 30 | 1419.2 | 779 |
| *consolidation* | 54 | 30 | 570.2 | 792.5 |
| *consolidation* | 54 | 30 | 2374.2 | 1609.4 |
| *consolidation* | 57.6 | 32 | 123.1 | 684.8 |
| *consolidation* | 54 | 27 | 70.7 | 1155.6 |
| *consolidation* | 59.4 | 33 | 55.1 | 1568.5 |
| *consolidation* | 54 | 30 | 84.7 | 681.8 |
| *palliative thoracic RT* | 38 | 19 | 2407 | 1464.6 |
| *consolidation* | 60 | 30 | 125 | 1004.8 |
| *consolidation* | 60 | 30 | 37.5 | 546.6 |
| *consolidation* | 54 | 18 | 246 | 505.6 |
| *consolidation* | 60 | 30 | 798.8 | 1444 |
| *consolidation* | 66 | 33 | 1026.1 | 1069.4 |
| *consolidation* | 50 | 10 | 38.1 | 266.9 |
| *consolidation* | 54 | 27 | 1119.6 | 871.4 |
| *consolidation* | 50 | 10 | 25.3 | 424.4 |
| *consolidation* | 60 | 30 | 286.6 | 887.9 |
| *consolidation* | 50 | 25 | 369.3 | 948.4 |
| *consolidation* | 60 | 30 | 447.1 | 828.3 |
| *consolidation* | 50 | 25 | 355.3 | 1110.7 |
| *consolidation* | 60 | 30 | 414.4 | 731.7 |
| *consolidation* | 60 | 30 | 124 | 879.7 |
| *consolidation* | 50 | 25 | 1375.2 | 1361.7 |
| *consolidation* | 60 | 30 | 1344.4 | 1006.1 |
| *consolidation* | 60 | 30 | 581.9 | 977.6 |
| *consolidation* | 60 | 30 | 457 | 909.5 |
| *palliative thoracic RT* | 40 | 20 | 747.6 | 822.9 |
| *consolidation* | 50 | 25 | 330.6 | 834.7 |
| *consolidation* | 60 | 30 | 1529.1 | 1602.4 |
| *consolidation* | 60 | 30 | 647.9 | 1185.2 |
| *consolidation* | 60 | 30 | 859.4 | 1540.9 |
| *consolidation* | 60 | 30 | 1137.3 | 993.8 |
| *consolidation* | 48 | 24 | 48.3 | 465.8 |
| *consolidation* | 50 | 25 | 379 | 959.7 |
| *consolidation* | 45 | 15 | 626.5 | 406.8 |
| *consolidation* | 60 | 30 | 1025.3 | 1651.8 |
| *consolidation* | 66 | 22 | 67.2 | 857.3 |
| *consolidation* | 58 | 24 | 507.3 | 1271.3 |
| *palliative thoracic RT* | 45 | 15 | 721.3 | 287.9 |
| *palliative thoracic RT* | 40 | 20 | 1465.2 | 954.2 |
| *consolidation* | 50 | 25 | 384.7 | 940.6 |
| *consolidation* | 60 | 30 | 2583.1 | 1868.5 |
| *consolidation* | 50 | 20 | 1385.9 | 584.4 |
| *consolidation* | 60 | 30 | 814.6 | 853.8 |
| *consolidation* | 55.8 | 31 | 703.1 | 1039.2 |
| *consolidation* | 50 | 25 | 1252.8 | 1052 |
| *consolidation* | 52 | 26 | 382.1 | 597.5 |
| *consolidation* | 54 | 27 | 1116.9 | 1260.9 |
| *consolidation* | 56 | 28 | 88.5 | 539.5 |
| *consolidation* | 56 | 28 | 1129.3 | 1039 |
| *consolidation* | 56 | 7 | 7.6 | 289.5 |
| *palliative thoracic RT* | 25 | 5 | 370.3 | 173.8 |
| *consolidation* | 56 | 28 | 505.5 | 631.4 |
| *consolidation* | 60 | 30 | 216.7 | 1110.5 |
| *consolidation* | 54 | 27 | 149.2 | 957.5 |
| *consolidation* | 60 | 30 | 1601.2 | 2055.8 |
| *palliative thoracic RT* | 40 | 10 | 2688.9 | 1326.1 |
| *palliative thoracic RT* | 40 | 20 | 1987.5 | 999.1 |
| *consolidation* | 60 | 30 | 1026.2 | 477.2 |
| *consolidation* | 60 | 12 | 715.4 | 602.6 |
| *consolidation* | 60 | 20 | 1965 | 861.2 |
| *palliative thoracic RT* | 39 | 13 | 1009 | 882.8 |
| *consolidation* | 54 | 18 | 14.3 | 728.5 |
| *palliative thoracic RT* | 30 | 7 | 28.8 | 213.7 |
| *palliative thoracic RT* | 30 | 7 | 235.1 | 351.4 |
| *consolidation* | 60 | 15 | 1683.5 | 1266.3 |
| *consolidation* | 60 | 30 | 595.9 | 719.3 |
| *palliative thoracic RT* | 30 | 7 | 1252.8 | 1052 |
| *palliative thoracic RT* | 24 | 8 | 1116.9 | 1260.9 |
| *consolidation* | 48 | 12 | 88.5 | 539.5 |
| *consolidation* | 50 | 4 | 1129.3 | 1039 |
| *consolidation* | 60 | 30 | 1425.6 | 845.7 |
| *consolidation* | 54 | 22 | 370.3 | 173.8 |
| *consolidation* | 60 | 30 | 1120.5 | 620.4 |
| *consolidation* | 50 | 20 | 765.4 | 400.3 |
| *consolidation* | 56 | 28 | 1320.7 | 750.2 |
| *consolidation* | 48 | 18 | 600.2 | 310.4 |
| *consolidation* | 58 | 29 | 1400.3 | 821.5 |
| *consolidation* | 53 | 25 | 970.4 | 564.1 |
| *consolidation* | 51 | 17 | 7.6 | 289.5 |

RT, radiotherapy; Gy, Gray; MHD, mean heart dose; MLD,mean lung dose; cGy,centigray;
